# Considerable scatter in the relationship between left atrial volume and pressure in heart failure with preserved left ventricular ejection fraction

**DOI:** 10.1038/s41598-019-56581-x

**Published:** 2020-01-09

**Authors:** Shiro Hoshida, Tetsuya Watanabe, Yukinori Shinoda, Tomoko Minamisaka, Hidetada Fukuoka, Hirooki Inui, Keisuke Ueno, Takahisa Yamada, Masaaki Uematsu, Yoshio Yasumura, Daisaku Nakatani, Shinichiro Suna, Shungo Hikoso, Yoshiharu Higuchi, Yasushi Sakata, Osaka CardioVascular Conference (OCVC) Investigators, Shunsuke Tamaki, Masatake Fukunami, Takaharu Hayashi, Yasuharu Takeda, Masaharu Masuda, Mitsutoshi Asai, Toshiaki Mano, Hisakazu Fuji, Yoshihiro Takeda, Yoshiyuki Nagai, Shizuya Yamashita, Yusuke Nakagawa, Shuichi Nozaki, Haruhiko Abe, Yasunori Ueda, Yukihiro Koretsune, Kunihiko Nagai, Masamichi Yano, Masami Nishino, Jun Tanouchi, Takatsugu Segawa, Shinji Hasegawa, Syouhei Yoshima, Minoru Ichikawa, Yoshiyuki Kijima, Eisai Rin, Masahiro Izumi, Hiroyoshi Yamamoto, Hiroyasu Kato, Kazuhiro Nakatani, Hisatoyo Hiraoka, Keiji Hirooka, Mayu Nishio, Takahiro Yoshimura, Yuji Okuyama, Tatsuya Sasaki, Akihiro Tani, Yasushi Okumoto, Hideharu Akagi, Yasunaka Makino, Katsuomi Iwakura, Yuzuru Takano, Nagahiro Nishikawa, Takashi Kitao, Hideyuki Kanai, Wataru Shioyama, Mikio Mukai, Masashi Fujita, Koichiro Harada, Osamu Nakagawa, Ryo Araki, Takayuki Yamada, Fusako Sera, Kei Nakamoto, Yasumasa Tsukamoto, Toshinari Onishi, Hidetaka Kioka, Tomohito Ohtani, Hiroya Mizuno, Takayuki Kojima, Tomoharu Dohi, Akito Nakagawa, Hirota Kida, Oeun Bolrathanak, Toshihiro Takeda, Yasushi Matsumura

**Affiliations:** 1 Department of Cardiovascular Medicine, Yao Municipal Hospital, Yao, Japan; 2 Division of Cardiology, Osaka General Medical Center, Osaka, Japan; 3 Department of Cardiovascular Medicine, National Hospital Organization Osaka National Hospital, Osaka, Japan; 4 Department of Cardiovascular Medicine, Amagasaki Chuo Hospital, Amagasaki, Japan; 5 Department of Cardiovascular Medicine, Osaka University Graduate School of Medicine, Suita, Japan; 6 Department of Cardiovascular Medicine, Osaka Police Hospital, Osaka, Japan; 7 Kansai Rosai Hospital, Amagasaki, Japan; 8 Kobe Ekisaikai Hospital, Kobe, Japan; 9 Rinku General Medical Center, Izumisano, Japan; 10 Kawanishi City Hospital, Kawanishi, Japan; 11 Ikeda Municipal Hospital, Ikeda, Japan; 12 Osaka Rosai Hospital, Sakai, Japan; 13 Japan Community Health Care Organization Osaka Hospital, Osaka, Japan; 14 Higashiosaka City Medical Center, Higashiosaka, Japan; 15 Kawachi General Hospital, Higashiosaka, Japan; 16 Kinki Central Hospital, Itami, Japan; 17 Japan Community Health Care Organization, Osaka Minato Central Hospital, Osaka, Japan; 18 Sumitomo Hospital, Osaka, Japan; 19 Saiseikai Senri Hospital, Suita, Japan; 20 National Hospital Organization Osaka Minami Medical Center, Kawachinagano, Japan; 21 Kano General Hospital, Osaka, Japan; 22 Kinan Hospital, Tanabe, Japan; 23 Hyogo Prefectural Nishinomiya Hospital, Nishinomiya, Japan; 24 Sakurabashi Watanabe Hospital, Osaka, Japan; 25 Japan Community Health Care Organization, Hoshigaoka Medical Center, Hirakata, Japan; 26 NTT West Osaka Hospital, Osaka, Japan; 27 Minoh City Hospital, Minoh, Japan; 28 Osaka International Cancer Institute, Osaka, Japan; 29 Suita Municipal Hospital, Suita, Japan; 30 Toyonaka Municipal Hospital, Toyonaka, Japan; 31 Otemae Hospital, Osaka, Japan

**Keywords:** Cardiology, Applied physics

## Abstract

The index for a target that can lead to improved prognoses and more reliable therapy in each heterogeneous patient with heart failure with preserved ejection fraction (HFpEF) remains to be defined. We examined the heterogeneity in the cardiac performance of patients with HFpEF by clarifying the relationship between the indices of left atrial (LA) volume (LAV) overload and pressure overload with echocardiography. We enrolled patients with HFpEF (N = 105) who underwent transthoracic echocardiography during stable sinus rhythm. Relative LAV overload was evaluated using the LAV index or stroke volume (SV)/LAV ratio. Relative LA pressure overload was estimated using E/e’ or the afterload-integrated index of left ventricular (LV) diastolic function: diastolic elastance (Ed)/arterial elastance (Ea) ratio = (E/e’)/(0.9 × systolic blood pressure). The logarithmic value of the N-terminal pro-brain natriuretic peptide was associated with SV/LAV (r = −0.214, p = 0.033). The pulmonary capillary wedge pressure was positively correlated to Ed/Ea (r = 0.403, p = 0.005). SV/LAV was negatively correlated to Ed/Ea (r = −0.292, p = 0.002), with no observed between-sex differences. The correlations between the LAV index and E/e’ and Ed/Ea and between SV/LAV and E/e’ were less prominent than the abovementioned relationships. SV/LAV and Ed/Ea, showing relative LAV and LA pressure respectively, were significantly but modestly correlated in patients with HFpEF. There may be considerable scatter in the relationships between these indices, which could possibly affect the selection of medications or efforts to improve the prognoses of patients with HFpEF.

## Introduction

The heterogeneity in the cardiac structure and function of patients with heart failure with preserved ejection fraction (HFpEF) is well known^1–3^. However, the index for a target that can lead to improved prognoses and more reliable therapy in a heterogeneous patient population remains to be defined. Early works have suggested that E/e’ could be used to reliably estimate the left ventricular (LV) filling pressure in the clinical setting of diastolic heart failure (HF)^4,5^. The correlations between E/e’ and direct left atrial (LA) pressure or pulmonary capillary wedge pressure (PCWP) are significant in a stable state^6–8^. LV diastolic elastance, expressed as Ed = (E/e’)/stroke volume (SV)^9^ or (E/e’)/LV end-diastolic volume^10^, and arterial elastance, expressed as Ea = (0.9 × systolic blood pressure)/SV^9^, are both higher in women than in men under stable conditions^9,10^. We previously reported that the Ed/Ea ratio is an index of LV diastolic function relative to afterload and is calculated as (E/e’)/(0.9 × systolic blood pressure), when Ed = (E/e’)/SV^11,12^. Therefore, Ed/Ea is not calculated by the parameters of cardiac volume, such as LA volume (LAV) and SV.

We recently reported that larger LAVs, relatively smaller LV volumes, and higher E/e’ and Ed/Ea ratios were observed in elderly women than in men with preserved LV ejection fraction (LVEF), in both those with and without HF^11–13^. In this study, we examined the extent to which the echocardiographic indices of volume and pressure overload in the left atrium are correlated with each other and the associated sex differences to elucidate the appropriate therapy for patients with HFpEF who have heterogeneous cardiac performance; these relationships could possibly affect the selection of medications or efforts to improve the prognoses of these patients. The LAV index (LAVI) or SV/LAV ratio was used as a marker of LAV overload, and the E/e’ or Ed/Ea ratio was used as a marker of LA pressure overload. As markers of myocardial function, we used the N-terminal pro-brain natriuretic peptide (NT-proBNP) levels and PCWP.

## Methods

We enrolled patients with HFpEF (N = 105, men/women 46/59) recruited from the PURSUIT HFpEF (Prospective, mUlticenteR, obServational stUdy of patIenTs with Heart Failure with Preserved Ejection Fraction) registry^13^. The PURSUIT HFpEF registry is from a prospective, multicentre observational study in which collaborating hospitals in the Osaka region of Japan record the clinical, echocardiographic, and outcome data of patients with HFpEF (UMIN-CTR ID: UMIN000021831). This study complied with the tenets of the Declaration of Helsinki, and the protocol (Osaka University Clinical Research Review Committee, R000024414) was approved by the ethics committee of each participating hospital (Ex. Ethics Committee of Yao Municipal Hospital, 2016-No.0006). Briefly, hospitalized patients with HF and an LVEF ≥ 50% were prospectively registered. All methods were performed in accordance with the relevant guidelines and regulations. All patients provided written informed consent to participate. We excluded patients with atrial fibrillation and/or considerable mitral or aortic valve disease.

Echocardiographic measurements were obtained according to the criteria of the American or European Society of Echocardiography during stable sinus rhythm^14,15^. In patients with HFpEF, echocardiography was performed while the patient was in stable condition before discharge. Volumetry was standardized using a modified Simpson’s method. The LAVI was calculated as the LAV divided by the body surface area. As markers of diastolic stiffness estimating the relative LA pressure overload, E/e’ and Ed/Ea (afterload-integrated diastolic elastance) were examined^11–13,16^. We evaluated the LAVI and LA ejection fraction (LAEF), which was calculated using SV/LAV, as relative markers of LAV overload. To ensure highly accurate measurements from the echocardiographic data, we performed short-course training sessions for echo technicians in each participating hospital several times^13^. The serum level of NT-proBNP and the estimated glomerular filtration rate (eGFR) were examined at the same time. A subset of the patients (N = 46) underwent right-heart catheterization and were examined for their PCWPs before discharge. The correlations among laboratory data, PCWP, and echocardiographic parameters were evaluated.

Continuous variables were expressed as means ± standard deviations, and categorical variables were presented as frequencies and percentages. The differences in categorical variables between the groups were compared using the chi-square test, and the differences in continuous variables between the groups were compared with Student’s t-test or Welch’s t-test, as appropriate. The correlations were tested using Pearson or Spearman coefficients, and the p-values were examined using regression analysis. Between-sex differences were evaluated using an interaction analysis. A p-value < 0.05 was considered significant.

## Results

The clinical and laboratory characteristics and echocardiographic data of the patients are shown in Table 1. The incidences of hypertension, diabetes mellitus, and dyslipidaemia were significantly different between the sexes. The LAVI was significantly larger in women than in men. However, there were no differences in the SV/LAV, E/e’, and Ed/Ea ratios between the sexes. The mean SV/LAV ratio was <1 in both men and women.

**Table 1:** Clinical and laboratory characteristics of patients with heart failure with preserved ejection fraction.

	All	Men	Women	*p- value*
	N = 105	N = 46	N = 59	
Age, years	78.5 ± 10.2	78.8 ± 10.7	78.3 ± 9.9	*0.791*
Body mass index, kg/m^2^	24.3 ± 5.0	25.0 ± 4.7	23.8 ± 5.2	*0.203*
Hypertension, n (%)	92 (88)	44 (96)	48 (81)	*0.028*
Diabetes mellitus, n (%)	43 (41)	24 (52)	19 (32)	*0.031*
Dyslipidaemia, n (%)	50 (48)	17 (37)	33 (56)	*0.041*
Systolic blood pressure, mmHg	120 ± 16	120 ± 15	120 ± 17	*0.767*
Diastolic blood pressure, mmHg	64 ± 12	63 ± 12	64 ± 14	*0.449*
Heart rate, bpm	70 ± 12	71 ± 12	69 ± 12	*0.424*
**Echocardiographic data**				
LAVI, mL/m^2^	47.6 ± 24.2	41.8 ± 13.8	52.1 ± 29.3	*0.030*
LVEDVI, mL/m^2^	59.3 ± 22.2	61.8 ± 24.4	57.3 ± 20.3	*0.299*
SVI, mL/m^2^	36.0 ± 12.8	36.2 ± 12.4	35.8 ± 13.1	*0.885*
SV/LAV	0.87 ± 0.38	0.92 ± 0.34	0.83 ± 0.41	*0.208*
LVEF, %	60.9 ± 6.9	59.5 ± 6.7	62.0 ± 7.0	*0.068*
LVMI, g/m^2^	113 ± 34	116 ± 34	110 ± 34	*0.381*
E/e’	14.4 ± 5.7	13.2 ± 3.8	15.4 ± 6.8	*0.051*
Ed/Ea, /mmHg	0.136 ± 0.058	0.123 ± 0.041	0.145 ± 0.068	*0.060*
**Laboratory data**				
Haemoglobin, g/dL	11.1 ± 1.8	11.4 ± 1.9	10.8 ± 1.6	*0.053*
eGFR, mL-min^−1^−1.73 m^−2^	41.1 ± 21.7	44.9 ± 22.8	38.2 ± 20.6	*0.117*
NT-proBNP, pg/mL	2,192 ± 4,017	1,724 ± 3,251	2,598 ± 4,632	*0.282*

Data are the mean ± standard deviation or number of patients (%).

The p-values represent the comparison of data between men and women.

LAVI, left atrial volume index; LVEDVI.

left ventricular end-diastolic volume index;

SVI, stroke volume index; LVEF, left ventricular ejection fraction;

LVMI, left ventricular mass index; Ed/Ea, diastolic elastance/arterial elastance;

eGFR, estimated glomerular filtration rate;

NT-proBNP, N-terminal pro-brain natriuretic peptide.

**Figure 1:**
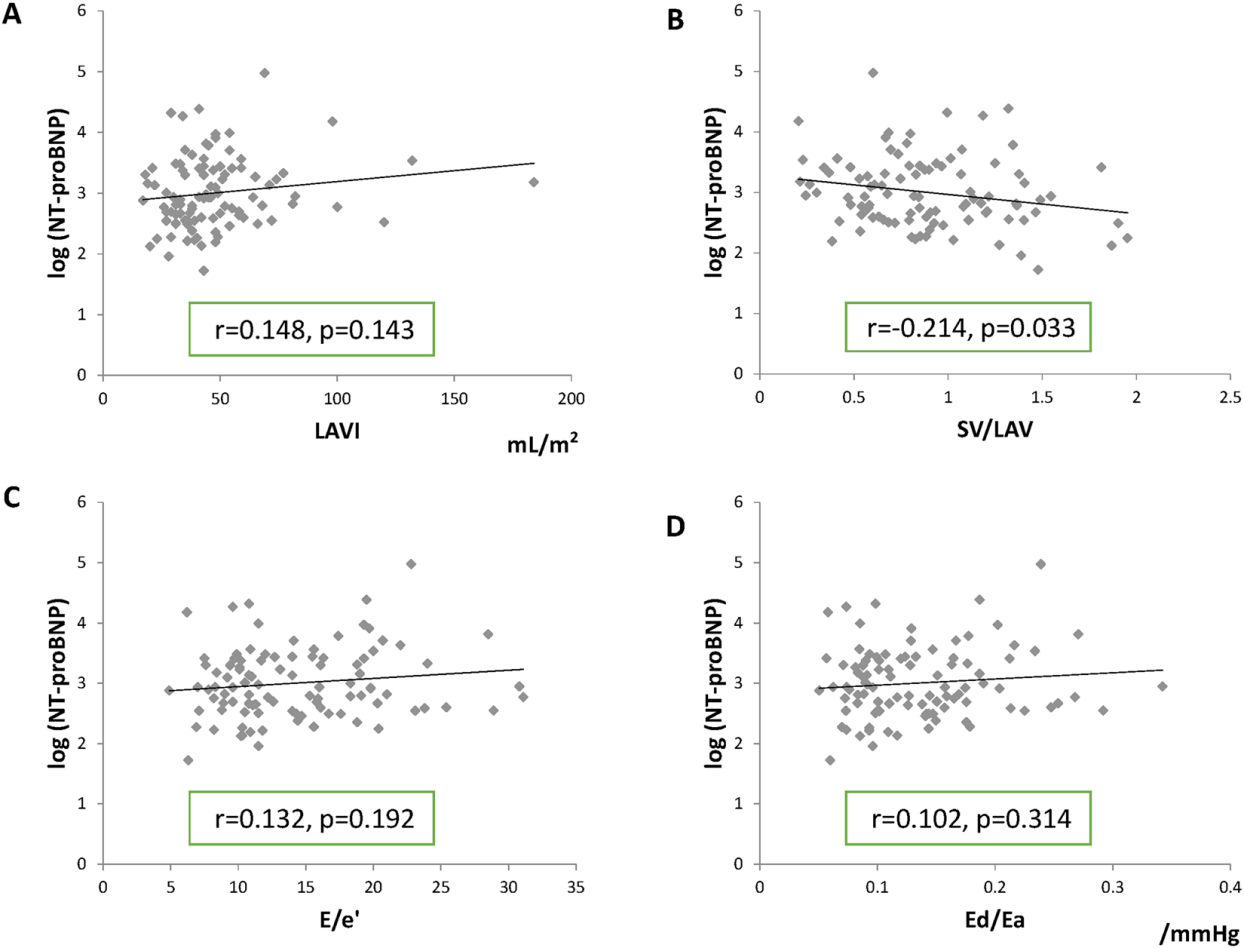
Correlations between the logarithmic value of serum N-terminal pro-brain natriuretic peptide (NT-proBNP) and several echocardiographic parameters (**A**–**D**) in patients with heart failure with preserved left ventricular ejection fraction before discharge. The logarithmic value of NT-proBNP was modestly associated with the stroke volume (SV)/left atrial volume (LAV) ratio (**B**), but not with the LAV index (LAVI, **A**), E/e’ (**C**), or diastolic elastance/arterial elastance ratio (Ed/Ea, **D**).

**Figure 2:**
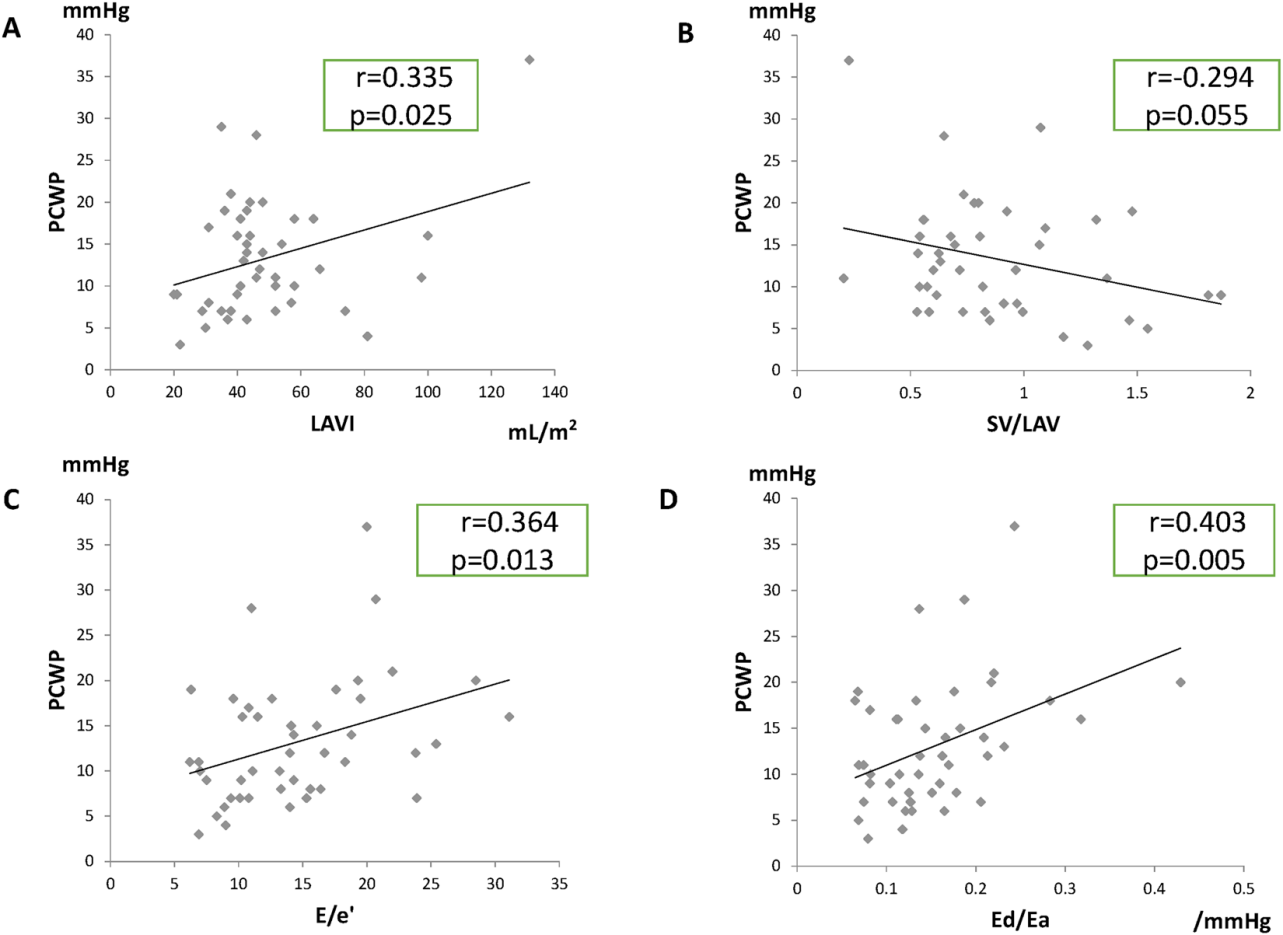
Correlations between pulmonary capillary wedge pressure (PCWP) and echocardiographic parameters (**A–D**) in patients with heart failure with preserved left ventricular ejection fraction before discharge. Significant correlations were observed between PCWP and the left atrial volume (LAV) index (LAVI, **A**), E/e’ (**C**), or the diastolic elastance/arterial elastance ratio (Ed/Ea, **D**) but not between PCWP and the stroke volume (SV)/LAV ratio (**B**).

The logarithmic value of NT-proBNP was modestly associated with SV/LAV (r = −0.214, p = 0.033) but not with LAVI (r = 0.148, p = 0.143), E/e’ (r = 0.132, p = 0.192), or Ed/Ea (r = 0.102, p = 0.314) (Fig. 1). Although not shown, a significant negative correlation was observed between the logarithmic value of NT-proBNP and eGFR (r = −0.527, p < 0.001). Modest positive correlations were observed between PCWP and LAVI (r = 0.335, p = 0.025), E/e’ (r = 0.364, p = 0.013) and Ed/Ea (r = 0.403, p = 0.005) (Fig. 2). No significant correlations were observed between Ed/Ea and pulmonary artery systolic pressure or right atrial mean pressure (data not shown).

**Figure 3:**
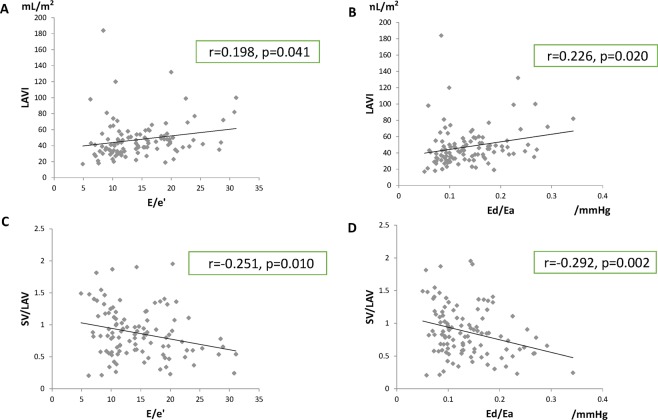
Relationship between the indices of volume and pressure in the left atrium. Modest correlations were observed between the left atrial volume (LAV) index (LAVI) and E/e’ as well as between the stroke volume (SV)/LAV ratio and the diastolic elastance (Ed)/arterial elastance (Ea) ratio (**A–D**). Among these, the correlation between the SV/LAV and the Ed/Ea ratio was more significant (**D**).

**Figure 4:**
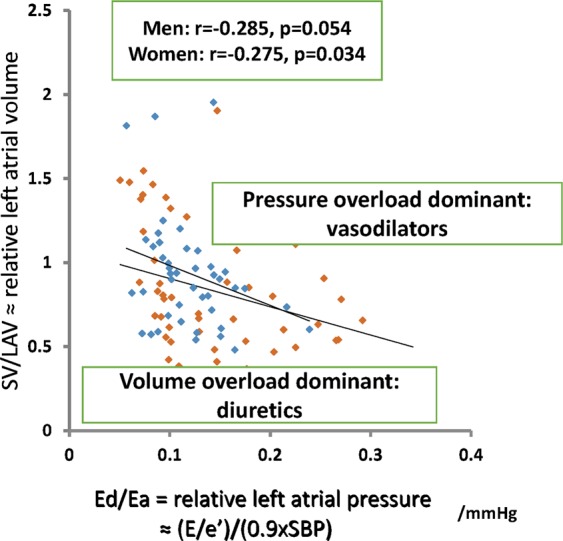
Left atrial (LA) pressure-volume relationship in patients with heart failure with preserved left ventricular ejection fraction. The vertical axis represents the stroke volume (SV)/LA volume (LAV) ratio, which shows the relative volume of the left atrium. The horizontal axis represents the diastolic elastance (Ed)/arterial elastance (Ea) ratio = (E/e’)/(0.9 × systolic blood pressure [SBP]), which shows the relative pressure in the left atrium. No sex differences in the relationship were observed. Patients represented in the lower left of the regression line may have volume overload of the left atrium, and volume reduction therapy such as diuretics may be useful in these patients. Patients represented in the upper right of the regression line may have pressure overload of the left atrium, and vasodilation therapy may be effective in these patients to avoid readmission. The blue circles represent data for men, and the orange circles represent data for women.

When we examined the relationship between the indices of LA volume and pressure overload, modest correlations were observed between LAVI and E/e’ as well as between SV/LAV and Ed/Ea (Fig. 3). Among these, the correlation between SV/LAV and Ed/Ea was more significant (r = −0.292, p = 0.002). No significant differences were observed in the correlations between sexes (Fig. 4) or between patients with and without diabetes mellitus (data not shown) (p interaction > 0.05). The correlations between the E/A ratio and LAVI (r = 0.119, p = 0.257) and SV/LAV (r = −0.161, p = 0.123) were less prominent than those between SV/LAV and Ed/Ea in patients with HFpEF.

## Discussion

Echocardiography can be a powerful tool for the diagnosis of diastolic dysfunction and elevated LV filling pressure^17,18^. The structure and function of the left atrium most closely reflect haemodynamic stress and remodelling in HFpEF patients^19^. LAEF usually indicates LA emptying functions^20,21^. However, we consider SV/LAV to be a negative index of LA volume overload that represents another aspect of LAEF under the conditions of preserved LVEF, and a modestly significant correlation was observed between NT-proBNP and the SV/LAV ratio. The SV/LAV and Ed/Ea ratios were significantly, but not closely, correlated in patients with HFpEF. No between-sex differences were observed in the correlation. When we examined the correlation between PCWP and echocardiographic parameters in patients with HFpEF before discharge, Ed/Ea had the most positive correlation with PCWP. Pulmonary congestion may occur under high Ed/Ea conditions, as observed in patients with HFpEF.

The correlation between Ed/Ea and SV/LAV may reflect indirect signs of relative pressure-volume relationships in the left atrium regardless of sex (Fig. 4). As the mechanisms of HFpEF onset are heterogeneous, there may be considerable scatter in the relationship between Ed/Ea and SV/LAV. In other words, the absence of a close correlation between the indices of volume and pressure overload in the left atrium may indicate that the worsening of one factor related to overload is adequate for HF to manifest in patients with HFpEF. Although both LA volume and pressure overload may affect the onset of HF in general, LA volume overload may be essential to precipitating the onset of HF in a subset of patients (volume reduction therapy is needed in these patients). In contrast, LA pressure overload may be an important factor in evoking the onset of HF in another subset of patients (in these patients, vasodilation therapy is effective in avoiding a volume shift to the third space of the body, which can result in readmission). A more accurate approach for HFpEF patient classification could define different therapeutic options, and more individualized treatment strategies could be provided to the different subsets. The Ed/Ea and SV/LAV ratios are novel echocardiographic parameters that may be useful for the selection of medical agents (Fig. 4) and for predicting the prognosis of patients with HFpEF. The large-scale, prospective PURSUIT HFpEF registry is continuously updated to clarify the differences in clinical outcomes (such as the incidence of readmission for HF and mortality) in relation to various parameters of cardiac volume and diastolic function (including SV/LAV and Ed/Ea), as well as in medications, among patients with HFpEF.

## Limitations

First, not all patients with HFpEF had invasive data, such as PCWP, collected before discharge. Second, we focused only on the left atrium but not on ventriculo-arterial coupling or right-sided heart function, which might be central in the pathophysiology of HFpEF. Because the Ed/Ea ratio, a marker of LA pressure overload, is an afterload-integrated index calculated as (E/e’)/(0.9 × systolic blood pressure) and the LV dimension and LVEF are within the normal ranges for patients with HFpEF, the relationship between SV/LAV and Ed/Ea may indirectly reflect ventriculo-arterial coupling. Third, we could not exclude the potential for measurement bias among facilities or investigators because the echocardiography evaluations were performed locally. Finally, we could not discuss the echocardiographic parameters of patients with atrial fibrillation.

## Conclusion

The SV/LAV and Ed/Ea ratios, which represent volume and pressure in the left atrium, were significantly but modestly correlated in patients with HFpEF. The considerable scatter in the relationship may affect the selection of medications or efforts to improve the prognoses of patients with HFpEF.
